# Impact of an Anti-Microbial Stewardship Program on Targeted Antimicrobial Therapy in a Tertiary Care Health Care Institute in Central India

**DOI:** 10.7759/cureus.18517

**Published:** 2021-10-05

**Authors:** Rahul Garg, Gyanendra Singh, Shweta Kumar, Mamta Verma, Lily Podder, Vaibhav Ingle, Abhishek Singhai, T Karuna, Saurabh Saigal, Kamini Walia, Sagar Khadanga

**Affiliations:** 1 General Medicine, All India Institute of Medical Sciences, Bhopal, IND; 2 Community and Family Medicine, All India Institute of Medical Sciences, Bhopal, IND; 3 Nursing, College of Nursing, All India Institute of Medical Sciences, Bhopal, IND; 4 Critical Care, All India Institute of Medical Sciences, Bhopal, IND; 5 Epidemiology and Communicable Diseases, Indian Council of Medical Research, New Delhi, IND

**Keywords:** targeted therapy, point prevalence survey (pps), bacterial culture rate, amsp, antimicrobial stewardship program

## Abstract

Introduction

Antimicrobial resistance (AMR) has become a global pandemic. In order to identify this menace, World Health Organisation (WHO) has developed the Global Action Plan on AMR (GAP AMR). Antimicrobial stewardship programs (AMSP) have been identified as a decisive tool for combating AMR. One of the most efficient measures of these programs has been the implementation of point prevalence surveys (PPS) of antibiotic usage and subsequent audit feedback. The present study was undertaken to identify the impact of AMSP on curtailing of empirical usage of antibiotics and the augmentation of targeted therapy.

Methods

It is an observational, cross-sectional study comprising 1396 patients. The microbiology culture details and anti-microbial-sensitivity results were recorded. Antibiotic prescriptions were recorded in each patient during their hospital stay.

Result

Out of 1396 patients treated over four quarters (Q1-Q4), 711 (50.9%) patients were on antibiotics, and among them, only 415 patients were subjected to any microbiological cultures with an overall bacterial culture rate (BCR) of 58.3%, and 296 patients (41.6%) were treated with antibiotics empirically without sending any samples for bacterial culture. There was a statistically significant rise in BCR from 47.3% in the first quarter to 77.6% in the fourth quarter. Sending specimens for blood culture increased significantly from 29.2% in Q1 to 37.6% in Q4. After receiving culture reports, 72.3% of cases continued with the same antibiotic, the antibiotic was changed in 19.9% of cases, and the antibiotic was stopped in 7.8% of cases.

Conclusion

There was a strong positive impact of AMSP in curtailment of empirical usage of antibiotics and augmenting targeted therapy as evidenced by the significant rise in BCR over Q1-Q4 PPS as well as a significant rise in ordering for blood culture over the same time period.

## Introduction

Inappropriate antimicrobial use is a significant determinant for the emergence of antimicrobial resistance (AMR) and multidrug-resistant organisms (MDROs). Increased antimicrobial misuse has been identified as a global concern. The economic consequences extend far beyond the human health sector to affect international travel and trade, potentially involving trillions of dollars. Identifying the urgency, World Health Organization (WHO) declared the Global Action Plan on AMR (GAP AMR) in 2015 [1]. In line with GAP AMR, India launched the National Action Plan on AMR (NAP AMR) in 2017 [2].

The implementation of antimicrobial stewardship programs (AMSP) in health care delivery centres has been advocated as one of the most critical and low-hanging targets to combat AMR. Many reputed bodies, including the Infectious Diseases Society of America (IDSA) and the Indian Council of Medical Research (ICMR), strongly recommended with moderate-quality of existing evidence for prescription audits and prospective feedback [3,4]. These measures can decrease the impact of AMR and drastically reduce out-of-pocket health-related expenditures in developing nations. Structured AMSP has improved antibiotic usage patterns, including prescription behaviour and patient outcomes [5-7]. Targeted antimicrobial therapy (TAMT) is the cornerstone of AMSP with the principles of reduction in empirical usage of antibiotics, scale-up of bacterial culture rate (BCR), and timely de-escalation of antibiotics.

Currently, AMSP is unstructured in India and practically non-existent in most government-run health care institutes [8]. Identifying the gap in India, ICMR initiated the Antimicrobial Resistance Surveillance and Research Network (AMRSN) and mentored regional sites in the country. The present study has been conducted at one of these sites to identify the impact of AMSP on TAMT over a one-year period. 

## Materials and methods

This was a cross-sectional study carried out at All India Institute of Medical Sciences (AIIMS) Bhopal, a tertiary government-run hospital, one of the regional centres of ICMR, which initiated the AMRSN. The study's primary objective was to enhance the targeted usage of antibiotics and diminish empirical antibiotic usage. All patients admitted to the hospital during the point prevalence survey (PPS) period were included in the study. The study design was approved by the Institutional Human Ethics Committee of All India Institute of Medical Sciences, Bhopal (Ref: IHEC-LOP/2018/EF0080). Individual consent was not obtained. The study was conducted with prior approval of the Institutional Human Ethics Committee (IHEC) AIIMS Bhopal and funding from ICMR. 

Point prevalence survey (PPS) was conducted on pre-fixed dates. These PPS were conducted at quarterly intervals over the study period of one year. Data of these four PPS have been taken into account. The four point prevalence surveys were conducted in Q1 (November 2018), Q2 (March 2019), Q3 (July 2019), and Q4 (Dec 2019). Data was collected from morning 8 AM to next day morning 8 AM on pre-determined dates by nursing officers posted at different areas and was later verified by the senior nursing officer of that area and the medical records department (MRD). The current survey focused on the identification of bacterial culture rate (BCR) and targeted antimicrobial therapy (TAMT). Microbiological samples were processed in the microbiology laboratory with the conventional and automated bacterial culture system and antimicrobial susceptibility testing (BacT Alert and Vitek II BioMérieux, Marcy-l'Étoile, France). Bacterial culture rate (BCR) was calculated as a fraction of patients in whom any sample was sent for microbiological culture among the total patients on antibiotics. 

All cases present in the hospital during the time mentioned above were included in the study, and hence, no formal sample size was calculated. 

Data were entered in Microsoft Excel and were summarized as frequencies and percentages for categorical variables. One decimal value was taken into account for the calculation of percentages. OpenEpi software (www.openepi.com) was used with a 95% confidence interval and R by C chi-square p-value of <0.05 were considered significant. 

## Results

Out of the 1396 total number of patients included in the study in four different quarters (Q1=280, Q2 =308, Q3=396, and Q4=412), 711 patients were on antibiotics (Q1=173, Q2=185, Q3 =192, and Q4=161). Overall bacterial culture rate (BCR) was 415 (58.3%). There was a statistically significant (chi-square p-value of < 0.001) increase in BCR over the four quarters (Q1 =47.3%, Q2=55.6%, Q3=54.6, Q4=77.6%) as seen in Table 1. 

**Table 1:** Consolidation in targeted antibiotic therapy across the point prevalence survey

Point prevalence survey	Total patients	No of patients on antibiotics, n (%)	Antibiotics started without sending culture, n (%)	Antibiotics started with sending culture, n (%)
Q1	280	173 (61.7)	91 (52.6)	82 (47.3)
Q2	308	185 (60.0)	82 (44.3)	103 (55.6)
Q3	396	192 (48.4)	87 (45.3)	105 (54.6)
Q4	412	161 (39.0)	36 (22.3)	125 (77.6)
Total	1396	711 (50.9)	296 (41.6)	415 (58.3)

Among the 711 patients exposed to any antibiotic (oral/intravenous), blood was the most common sample ordered (19.4%) for bacterial culture. The incidence of sending blood culture increased significantly (p=0.002) across the quarters of PPS (Q1=13.8%, Q2=18.9%, Q3=16.6%, and Q4=29.1%). Urine was the second most common sample sent for bacteriological culture, accounting for 12.7% of cases. Similarly, the percentage of other samples sent for bacteriological culture among the patients exposed to the antibiotic are as follows: respiratory secretion (6.3%), pus (9.4%), and body fluids (8.4%). Among the total samples sent for bacteriological culture (n=415), the overall sample distribution was blood (33.2%), urine (25.3%), pus (16.1%), respiratory secretions (10.8%), and body fluids (14.4%). The detailed spectrum of bacterial culture samples across the various PPS quarters is provided in Table 2.

**Table 2:** Spectrum and yield of bacterial culture specimens across the point prevalence survey across four quarters (Q1-Q4)

Patients on antibiotics	Blood		Urine		Respiratory		Pus		Body fluid		Total	
	Sample Sent	Culture Positivity	Sample Sent	Culture Positivity	Sample Sent	Culture Positivity	Sample Sent	Culture Positivity	Sample Sent	Culture Positivity	Sample Sent	Culture Positivity
Q1 (n=173)	24 (13.8)	11 (45.8)	24 (13.8)	9 (37.5)	9 (5.2)	7 (77.7)	14 (8.0)	9 (64.2)	11 (6.3)	8(72.7)	82 (47.3)	44 (53.7)
Q2 (n=185)	35 (18.9)	21 (60.0)	24 (12.9)	11 (45.8)	15 (8.1)	3 (20.0)	13 (7.0)	11 (84.6)	16 (8.6)	7 (43.7)	103 (55.6)	53 (50.4)
Q3 (n=192)	32 (16.6)	23 (71.8)	26 (13.5)	14 (53.8)	6 (3.1)	3 (50.0)	22 (11.4)	14 (63.6)	19 (9.8)	12 (63.1)	105 (54.6)	66 (62.9)
Q4 (n=161)	47 (29.1)	18 (38.2)	31 (19.2)	11 (35.4)	15 (9.3)	4 (26.6)	18 (11.1)	3 (16.6)	14 (8.6)	3 (21.4)	125 (77.6)	39 (31.2)
Total=711	138 (19.4)	73 (52.8)	105 (12.7)	45 (42.8)	45 (6.3)	17 (37.7)	67 (9.4)	37 (55.2)	60 (8.4)	30 (50.0)	415 (58.3)	202 (48.6)

The microbiological evidence of bacterial infection based on culture reports was observed in 202 (48.6%) cases. Overall, the pus culture positivity rate was the highest 37 (55.2%). The average culture-positive rates of blood, urine and respiratory samples were 52.8%, 42.8%, and 37.7%. The details of culture-positive rates of various samples across the quarter-wise PPS have been provided in Table 2. 

Out of 711 patients who were on antibiotics across the four PPS, 296 patients were exposed to antibiotics without sending any sample for bacterial culture. So, the overall empirical antibiotic usage was 296 (41.6%). This decrease in empirical usage of antibiotics among the antibiotic-exposed population was significant (chi-square p < 0.001). This trend of decrease in empirical usage of antibiotics was also significant even after taking into account the whole population of 1396 patients surveyed across the four PPS as the denominator (chi-square p < 0.001). After receiving the culture susceptibility report, 72.3% of cases continued with the same antibiotic, the antibiotic was changed in 19.9% of cases, and the antibiotic was stopped in 7.8% of cases. 

## Discussion

AMR and the subsequent emergence of MDRO is one of the greatest threats to humankind. Though the menace of AMR spares no country, developing nations like India seem to be maximally affected [8]. In a systematic review by Saleem et al. (2020), the highest use of broad-spectrum antimicrobials was seen in India [9]. 

Institutional AMSP has been advocated to cut edge in decreasing AMR and the subsequent emergence of MDRO [5-7]. Point prevalence study of antibiotic usage as part of AMSP has been identified as one of the most cost-effective measures to identify the antibiotic prescription pattern [6,7]. In this context, the present study was conducted to evaluate the impact of AMSP on the reduction of empirical usage of antibiotics and the augmentation of targeted antimicrobial therapy. 

The overall antibiotic consumption derived from the point prevalence study from our institute has been reported earlier and found to be 711 (50.9%) [10]. The overall bacterial culture rate (BCR) in our study is 58.3%. BCR in different studies is within 15% to 70.5% in various countries as can be seen in Table 3 [11-20].

**Table 3:** Bacterial culture rate (%) in various countries

Author	Year	Country	Bacterial culture rate (%)
Limato et al. [19]	2021	Indonesia	44.3
Amponsah et al. [20]	2021	Ghana	2.7
Gürtler et al. [18]	2019	Switzerland	69
Ciofi degli Atti et al. [13]	2019	Italy	61.2
Haldrup et al. [17]	2017	Denmark	15
Ren et al. [14]	2016	China	40.16
Zarb and Goossens [15]	2015	European countries	46.8
Li et al. [11]	2013	China	34.7
Ingram et al. [12]	2012	Australia	36
Curcio et al. [16]	2011	Latin America	70.5

The present study highlights the fact that over one year of initiation of AMSP, the BCR increased from 47.3% in the first quarter to 77.6% in the fourth quarter. This emphasizes the importance of running AMSP to decrease empirical antibiotic use and augmentation of microbiological targeted therapy. We could not find any other studies to compare our result of the significant increase in BCR with extended PPS. 

The overall percentage of various specimens sent for bacterial culture in our study was blood (33.2%), urine (25.3%), pus (16.1%), respiratory secretions (10.8%), and other body fluids (14.4%). Mohammad et al. (2020) in a study set in the southern part of India have documented blood and urine specimens constituting 95% of total samples (60% and 35% respectively); they might not have included enough surgical wards and intensive care units, which may explain the discrepancy with our study [21]. We could not find any global data of PPS mentioning the fraction of various specimens to compare with. In our study, the relative fraction of sending blood samples for bacterial culture increased over the Q1-Q4 of PPS. This finding reiterates the importance of PPS and AMSP. 

In our study, pus culture positivity was 55.2%. A similar result (61.8%) was documented by Khanam et al. (2018) from Bangladesh [22]. Blood culture positivity rate has usually been reported about 10% in most studies across the globe [23,24]. Our blood culture positivity rate was significantly high at a level of 52.8%. Our study observed that about 40% of patients were started on empirical antibiotics without sending any microbiological sample. As this result is almost similar to the study from the United Kingdom by Hamilton et al., it seems a similar trend is found all across the globe [25]. However, we observed that the empirical usage of antibiotics decreased significantly from 52.6% in the first quarter of PPS to 22.3% in the fourth quarter of PPS. This re-emphasizes that the practice of AMSP over a long period of time helps in the augmentation of targeted therapy and reduction in empirical therapy. We could not find any other study with which to compare our improvement in empirical usage of antibiotics over an extended PPS. After receiving the antimicrobial susceptibility report, we noticed that the same antibiotic was continued in about 70% of cases, and the antibiotic was changed only in about 20% of cases. Similar observations have been reported from India and outside. In a retrospective study over 10 months in a level 1 trauma centre in India, only 13.5% of antimicrobial prescriptions were based on culture reports [26]. In another antibiotic utilization study in Saudi Arabia, antimicrobial modification based on available culture results was performed in only 12% of patients [27]. These findings suggest that empirical antibiotic usage has been one of the major causes of AMR globally, and targeted therapies are the need of the hour.

Our study has a few limitations, namely that data were collected manually without the aid of e-platforms. Nursing officers were involved in data collection and compilation rather than doctors. Data of culture-negative sepsis patients were not captured.

## Conclusions

There was a strong positive impact of AMSP in curtailment of empirical usage of antibiotics and augmenting targeted therapy as evidenced by the significant rise in BCR over first to fourth quarter PPS and a significant rise in ordering for blood culture over first to fourth quarter PPS. This positive impact of curtailment of empirical usage of antibiotics resulted from simple and yet robust targets like quarterly PPS study and subsequent audit and feedback. It can be concluded that it is feasible to practice low-budget AMSP in government-run hospitals with a significant impact. Leadership quality, changing antibiotic prescription behaviour, and perseverance to decrease AMR is the need of the hour. 
